# Long‐term continuation of anti‐seizure medications after acute stroke

**DOI:** 10.1002/acn3.51440

**Published:** 2021-08-06

**Authors:** Vineet Punia, Ryan Honomichl, Pradeep Chandan, Lisa Ellison, Nicolas Thompson, Adithya Sivaraju, Irene Katzan, Pravin George, Chris Newey, Stephen Hantus

**Affiliations:** ^1^ Epilepsy Center Neurological Institute Cleveland Clinic Cleveland Ohio USA; ^2^ Department of Quantitative Health Sciences Lerner Research Institute Cleveland Clinic Cleveland Ohio USA; ^3^ Center for Outcomes Research and Evaluation Neurological Institute Cleveland Clinic Cleveland Ohio USA; ^4^ Comprehensive Epilepsy Center Department of Neurology Yale University New Haven Connecticut USA; ^5^ Cerebrovascular Center Neurological Institute Cleveland Clinic Cleveland Ohio USA

## Abstract

**Objective:**

To investigate the factors associated with the long‐term continuation of anti‐seizure medications (ASMs) in acute stroke patients.

**Methods:**

We performed a retrospective cohort study of stroke patients with concern for acute symptomatic seizures (ASySs) during hospitalization who subsequently visited the poststroke clinic. All patients had continuous EEG (cEEG) monitoring. We generated a multivariable logistic regression model to analyze the factors associated with the primary outcome of continued ASM use after the first poststroke clinic visit.

**Results:**

A total of 507 patients (43.4% ischemic stroke, 35.7% intracerebral hemorrhage, and 20.9% aneurysmal subarachnoid hemorrhage) were included. Among them, 99 (19.5%) suffered from ASySs, 110 (21.7%) had epileptiform abnormalities (EAs) on cEEG, and 339 (66.9%) had neither. Of the 294 (58%) patients started on ASMs, 171 (33.7%) were discharged on them, and 156 (30.3% of the study population; 53.1% of patients started on ASMs) continued ASMs beyond the first poststroke clinic visit [49.7 (±31.7) days after cEEG]. After adjusting for demographical, stroke‐ and hospitalization‐related variables, the only independent factors associated with the primary outcome were admission to the NICU [Odds ratio (OR) 0.37 (95% CI 0.15–0.9)], the presence of ASySs [OR 20.31(95% CI 9.45–48.43)], and EAs on cEEG [OR 2.26 (95% CI 1.14–4.58)].

**Interpretation:**

Almost a third of patients with poststroke ASySs concerns may continue ASMs for the long term, including more than half started on them acutely. Admission to the NICU may lower the odds, and ASySs (convulsive or electrographic) and EAs on cEEG significantly increase the odds of long‐term ASM use.

## Introduction

Acute symptomatic seizures (ASySs) occur in 4% to 15% of stroke patients.^1^, ^2^, ^3^ There is extensive literature debating anti‐seizure medication (ASM) use for primary prophylaxis of ASySs after stroke, especially in ICH patients.^4^, ^5^, ^6^, ^7^ In contrast, there are limited data on ASM management after ASySs in stroke patients.^8^ Noting that the use of ASMs after ASySs is common, the European Stroke Organization (ESO) proposes stopping them after the “acute” period.^8^ While ASySs are clearly defined as seizures within 7 days of stroke,^9^ the acute period for ASM use after ASySs remains undefined. Some authors recommend ASM use for as long as 4 years after stroke‐related ASySs.^10^

The use of continuous electroencephalogram (cEEG) monitoring reveals a higher frequency of ASySs in stroke patients than previously noted, because a large proportion are nonconvulsive.^11^, ^12^, ^13^ Additionally, cEEG reveals other epileptiform abnormalities (EAs) such as lateralized periodic discharges (LPDs), lateralized rhythmic delta activity (LRDA), and generalized periodic discharges (GPDs) in approximately 30% of the recordings.^14^ They are commonly noted after stroke and are associated with increased ASyS risk.^14^, ^15^ Experts recommend using ASMs for a month to up to a year in patients with LPDs, ASySs, or both.^16^ While almost all patients with electrographic ASySs are discharged from the hospital on ASMs,^17^ their duration of continuation remains poorly investigated, especially in the era of cEEG monitoring.

The use of ASMs after hospital discharge in stroke patients is not trivial. A vast majority of patients experience adverse effects on ASMs, including cognitive slowing, mood problems, and gait instability.^18^, ^19^, ^20^, ^21^ ASMs interact with other medications,^22^ and negatively affect the quality of life after stroke.^23^ Therefore, there is a need to better understand the long‐term use of ASMs in stroke patients. For stroke patients discharged on ASMs, the poststroke clinic visit provides an excellent opportunity to assess their continuing needs. Therefore, our study's primary aim is to investigate the long‐term continuation of ASMs beyond the first poststroke clinic visit and the predictors of this practice.

## Methods

After Institutional Review Board (IRB) approval, we conducted a single‐center, retrospective cohort study. The inclusion criteria for the study population were as follows: adults (≥18 years of age), diagnosed with acute stroke from 1 April 2012 to 31 March 2018, underwent cEEG monitoring in the acute period (≤7 days of stroke) due to ASySs concerns, and had at least one poststroke clinic visit. Patients with history of epilepsy and newly diagnosed epilepsy (seizures >7 days after stroke) were excluded.^24^ The study population was identified using three prospectively maintained patient databases: our internal acute stroke database, EEG database, and Cleveland Clinic Knowledge Program (KP) database, a previously described^25^ prospective registry of patient‐reported outcomes collected as part of standard care. Every acute brain injury patient with convulsive seizures or suspected nonconvulsive seizure/status epilepticus (NCS/NCSE; electrographic seizures) undergoes cEEG monitoring for at least 12 h at our institution. Combined, these patients were defined as having “ASySs concern.” Of note, our institution does not have a protocol to start all hemorrhage patients on ASM. The study population's inpatient and outpatient data were retrieved from the electronic medical record (EMR) and EEG database review.

Study variables include age, sex, stroke type [ischemic stroke (IS), subarachnoid hemorrhage (SAH), ICH], National Institutes of Health Stroke Scale Score (NIHSS) at presentation, cortical involvement by the stroke, convulsive seizure before cEEG, mental status at the start of cEEG (awake/lethargic vs. stuporous/coma), convulsive or electrographic seizures on cEEG (latter defined by Salzburg criteria^26^), EAs on cEEG (including isolated sharp waves,^27^ LPDs, GPDs, LRDA as defined by American Clinical Neurophysiology Society^28^), prior stroke history, neuro ICU (NICU) admission, discharging team (medicine/surgery, neurology, neurosurgery), discharge disposition (home vs. facility), type of provider during clinic visit [advanced practice providers (APP), neurologist, neurosurgeon], as well as ASM management during hospitalization, at discharge, and at the poststroke clinic visit. Patients were considered started on an ASM during hospitalization if they received two or more maintenance doses after the initial loading dose. The primary outcome was the continuation of patients on ASM beyond the poststroke clinic visit (long‐term ASM continuation). Patients with only dose reduction or deprescription of one of the multiple ASMs were also considered meeting the primary outcome.

### Statistical methods

We used descriptive statistics to summarize demographic/clinical characteristics. Frequency counts with percentages were used to present categorical data. Mean with standard deviation and median with inter‐quartile range (IQR, first quartile–third quartile) were used to present continuous variables, as appropriate. The association between predictor variables and the primary outcome variable was initially determined with univariate logistic regression models. Predictors associated with the outcome at least at the *p* < 0.05 level, as well as covariates deemed clinically important (e.g., stroke type, age, mental status) were added to a multivariable logistic regression model. For this model, we only included patients who were started on ASMs during the hospitalization, rather than the entire study cohort. Computations were performed in R, version 4.0.2.^29^

## Results

### Study cohort

**Figure 1:**
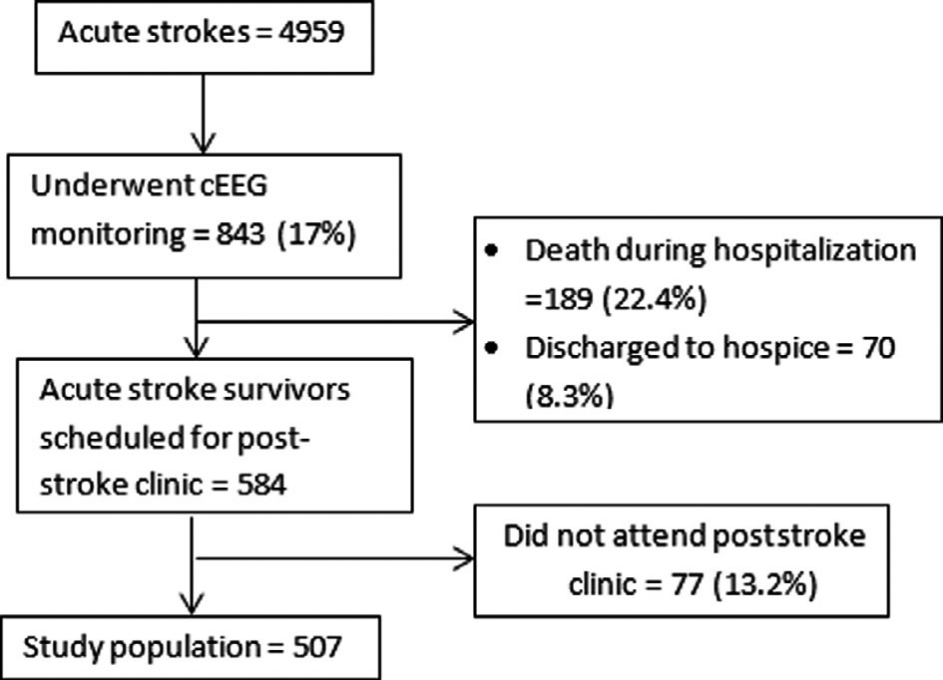
Study cohort flowchart for the study period (1 April 2012 to 31 March 2018). Percentages shown as proportion of the number of patients a step‐above in the flowchart.

A total of 507 patients met the inclusion criteria (study cohort; Fig. 1). There were 220 (43.4%) IS patients, 181 (35.7%) ICH patients, and the rest had aneurysmal SAH (106; 21%). None of the patients were on ASMs at the time of stroke. A total of 99 (19.5%) patients had ASySs, including 41 who were found to have seizures on cEEG. Among the latter, 37 (90.2%) had electrographic seizures only, and nine (22%) had suffered a pre‐cEEG convulsive ASySs as well. Additionally, 110 (21.7%) patients had EAs on cEEG monitoring. There were 339 (66.9%) patients who had neither ASySs nor EAs on cEEG. Table 1 provides details on the electro‐clinical, hospital admission, and clinic‐related variables of the study cohort overall and according to ASM use during the admission.

**Table 1:** Demographic and clinical characteristics of study population.

	Study cohort (*n* = 507)	ASM during admission (*n* = 294; 58%)	No ASM during admission (*n* = 213; 42%)	*p* values
Age (years)	62.0 (14.1)	60.8 (14.0)	63.7 (14.2)	0.023
NIHSS at admission^1^	8.8 (8.2)	9.0 (8.4)	8.5 (8.0)	0.503
Hospitalization duration (days)	12.4 (9.6)	13.7 (9.5)	10.5 (9.5)	<0.001
Time from hospital discharge until clinic visit (days)	37.0 (30.3)	38.5 (31.2)	35.8 (29.7)	0.331
Female	263 (51.9)	159 (54.1)	104 (48.8)	0.281
History of stroke, prior to admission	54 (10.7)	33 (11.2)	21 (9.9)	0.729
Stroke type				<0.001
Ischemic stroke	220 (43.4)	83 (28.2)	137 (64.3)	
ICH	181 (35.7)	119 (40.5)	62 (29.1)	
SAH	106 (20.9)	92 (31.3)	14 (6.6)	
Cortex involved	284 (56.0)	170 (57.8)	114 (53.5)	0.383
Initial mental status: Stupor/Coma	98 (19.3)	69 (23.5)	29 (13.6)	0.008
Patient admitted in NICU	375 (74.0)	245 (83.3)	130 (61.0)	<0.001
Discharging team				<0.001
Neurology	258 (51.0)	118 (40.3)	140 (65.7)	
Med/Surg	60 (11.9)	22 (7.5)	38 (17.8)	
Neurosurgery	188 (37.2)	153 (52.2)	35 (16.4)	
Seizures (convulsive or electrographic)^2^	99 (19.5)	97 (33.0)	2 (0.9)	<0.001
Convulsive seizure before cEEG (%)	67 (13.2)	65 (22.1)	2 (0.9)	<0.001
Epileptiform abnormalities on cEEG^3^	110 (21.7)	92 (31.3)	18 (8.5)	<0.001
Discharge destination: home	187 (36.9)	104 (35.4)	83 (39.0)	0.463
Seizure after discharge	8 (1.6)	8 (2.7)	0 (0.0)	0.037
Type of provider at follow‐up visit				<0.001
Neurologist	258 (50.9)	123 (41.8)	135 (63.4)	
APP	71 (14.0)	45 (15.3)	26 (12.2)	
Neurosurgeon	178 (35.1)	126 (42.9)	52 (24.4)	

Data presented as mean (standard deviation) or *N* (%).

^1^ Missing NIHSS = 106 (20.91%) patients.

^2^ Nine patients had seizures prior to the start of cEEG as well.

^3^ None of the patients had only GPDs.

In the study cohort, 294 (58.0%) were started on ASMs during the hospitalization. Compared to the patients not started on ASMs, they were more likely to have experienced an ICH (41% vs. 29%, *p* = 0.011) or an SAH (31% vs 7%; *p* < 0.0001) and less likely an IS (28% vs. 64%; *p* < 0.0001). Additionally, patients started on ASMs were more likely to be admitted into the NICU (83% vs 61%; *p* < 0.001) and had a longer hospitalization duration (13.68 ± 9.51 vs. 10.52 ± 9.54; *p* < 0.001). The two groups did not differ in terms of prior stroke history or the presenting NIHSS. As expected, patients started on ASM were more likely to have seizures (33% vs. 1%, *p* < 0.001), or show EAs on cEEG (31% vs. 9%, *p* < 0.001) (Table 1).

### ASM use

**Figure 2:**
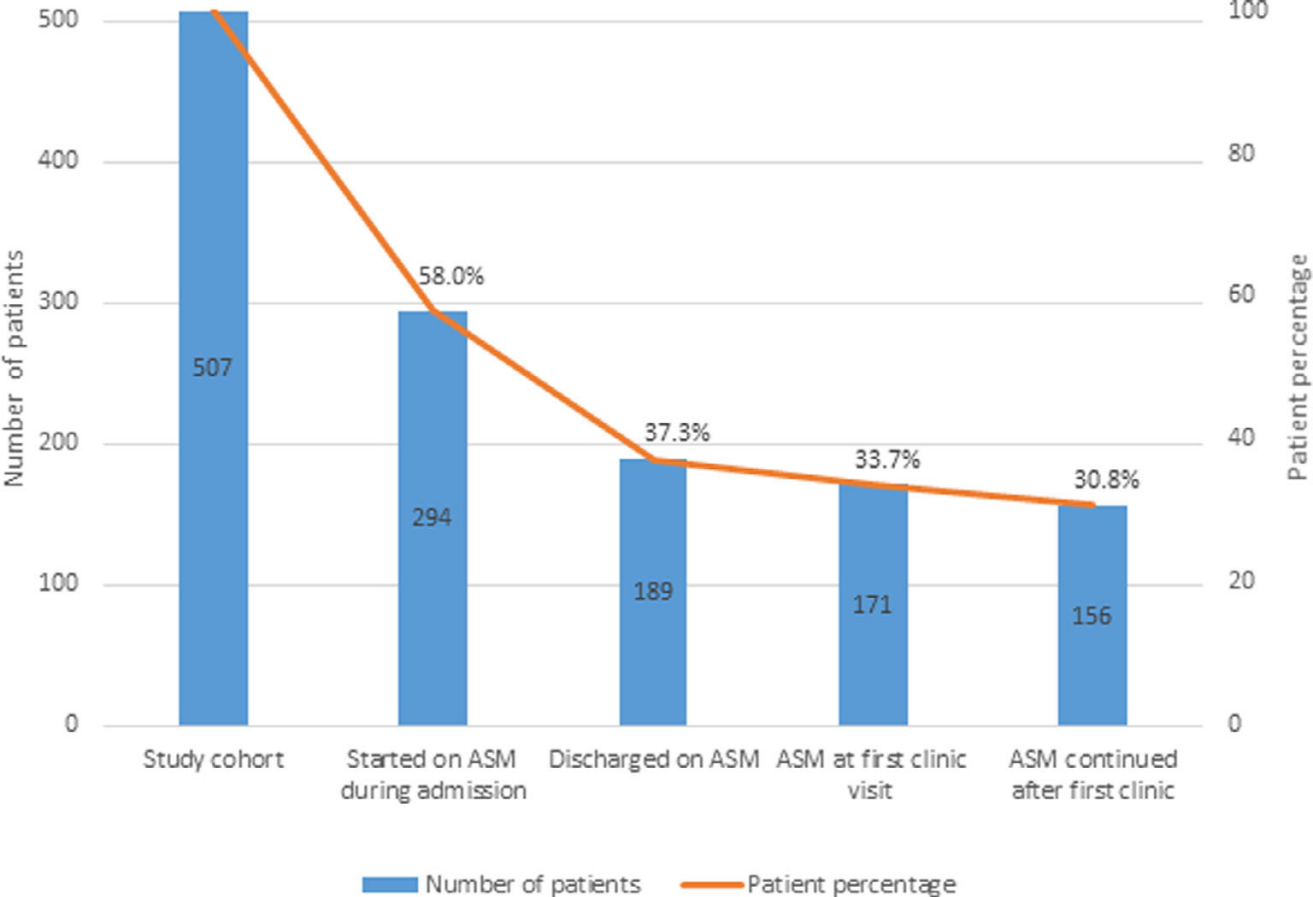
Study cohort and ASM use at various clinical care stages.

The ASM use at various clinical care stages is presented in Figure 2. Out of the 294 patients started on ASMs, 189 (37.3% of study cohort; 64.3% of patients started on ASM) were discharged on them. Among the patients discharged on ASMs, 171 (33.7%) were taking them at the first poststroke clinic visit, and 156 (30.8%) were continued on them after the first poststroke clinic visit. These 156 patients, who met the study's primary outcome, account for 82.5% of the patients discharged on ASMs and 53.1% of patients started on ASMs during hospitalization. For the patients started on ASMs during admission, its discontinuation was more likely to occur prior to hospital discharge [*n* = 105 (35.7%)] compared to after discharge [*n* = 33 (11.2%); *p* = 0.0075)].

Patients started on ASMs (*n* = 294) were taking a mean of 1.23 (±0.5) [media *n* = 1 (IQR = 1–1)] ASMs during hospitalization. Five patients required intravenous (IV) anesthetics for seizure control. Patients discharged on ASMs (*n* = 189) were prescribed a mean of 1.34 (±0.58) [media *n* = 1 (IQR = 1–2)] ASMs, which includes 18 patients on more than one ASM. At the clinic visit, patients taking ASMs (*n* = 171) were on a mean of 1.36 (±0.59) [media *n* = 1 (IQR = 1–2)] ASMs. Patients continued on ASMs (*n* = 156) were taking a mean of 1.1 (±0.36) [media *n* = 1 (IQR = 1–1)] ASMs.

**Figure 3:**
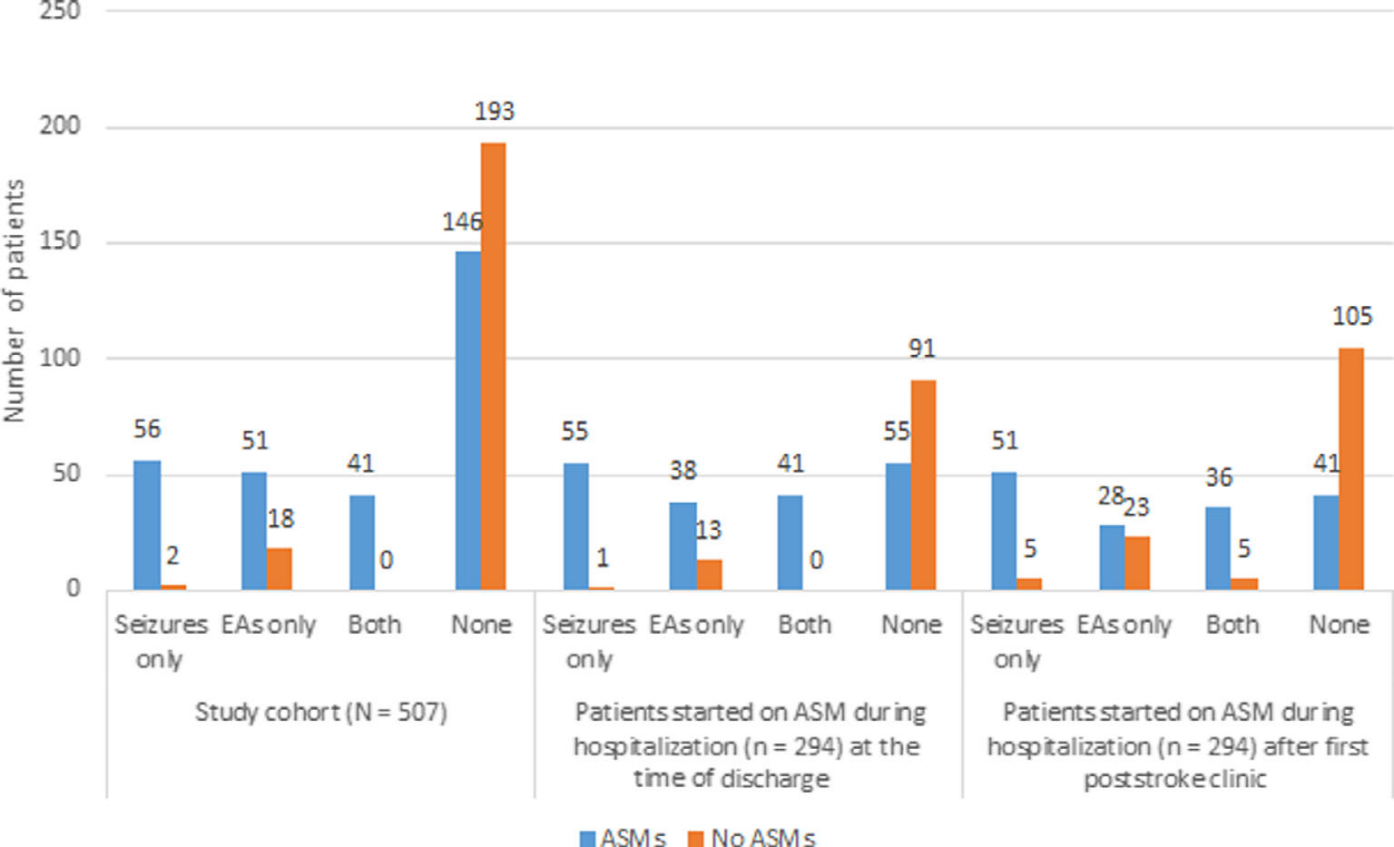
ASM use at various clinical care stages in relation to ASySs and EAs in the acute period.

**Figure 4:**
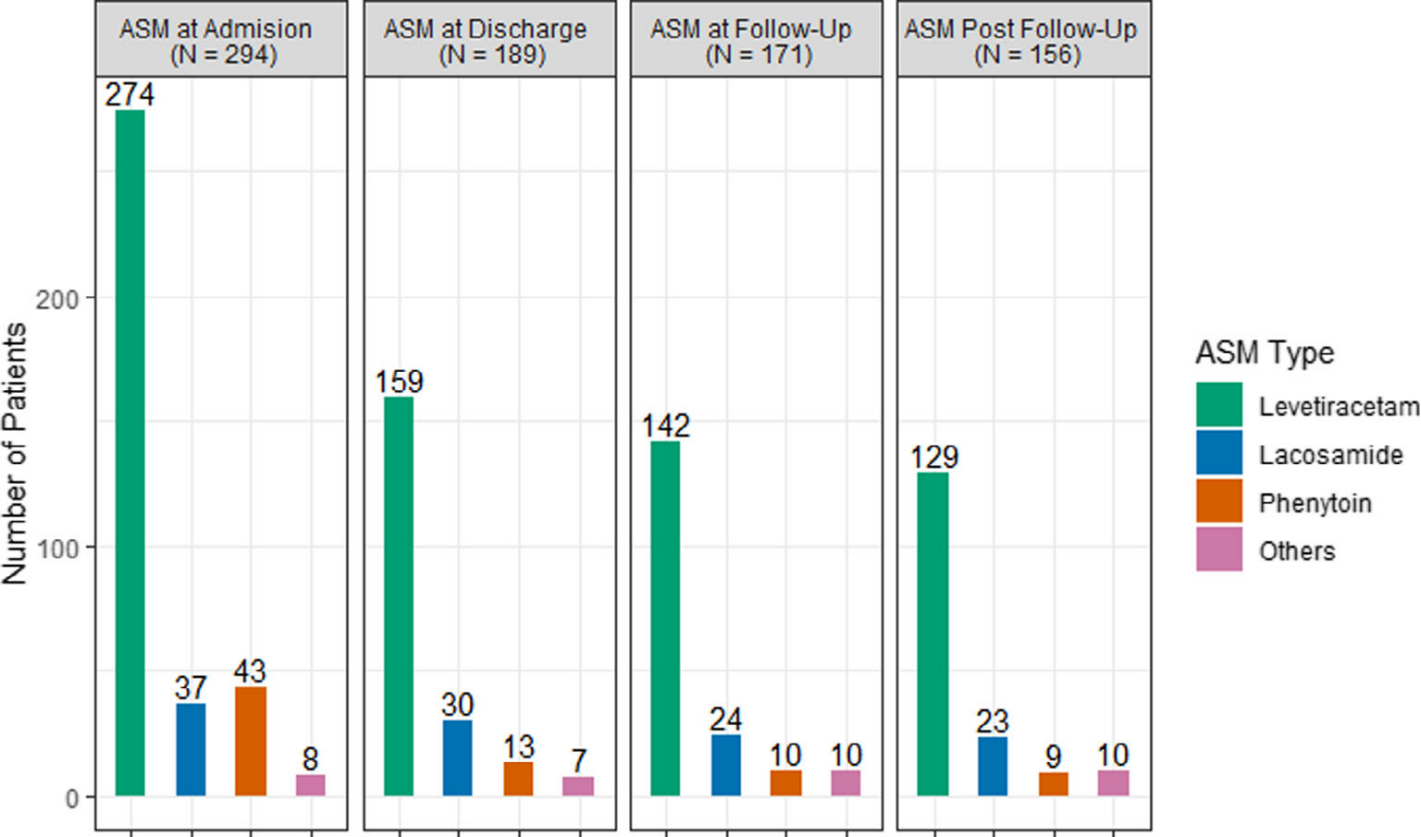
ASMs used in the study population at various clinical care stages. Other ASMs during admission: Valproate = 3, Topiramate = 2, Lamotrigine = 1; Other ASMs at time of discharge: Valproate = 3, Topiramate = 2, Lamotrigine = 2, Phenobarbital = 1; Other ASMs at and postclinic follow‐up: Valproate = 4, Topiramate = 1, Lamotrigine = 1, Phenobarbital = 2, Oxcarbazepine = 1, Zonisamide = 1.

Figure 3 provides the distribution of ASM use during hospitalization, at hospital discharge, and after the first poststroke visit in relation to the presence of ASySs and EAs. A total of 146 patients without ASySs or EAs were started on ASM, including 75 patients who underwent a neurosurgical procedure (Table S1). Of the 105 patients undergoing ASM discontinuation prior to hospital discharge, 91 (86.7%) did not have ASySs or EAs on cEEG. Among patients continued on ASMs beyond the first poststroke clinic visit (*n* = 156), 41 (26.3%) did not have ASySs or EAs on cEEG. All patients on multiple ASMs at the time of discharge were continued on ASMs beyond the first poststroke clinic visit, with only four undergoing deprescription of one ASM. The different ASMs used at various clinical care stages, from the admission to long‐term continuation after the clinic visit, are represented in Figure 4. Levetiracetam was, by far, the most commonly used ASM at all measured clinical care stages. Phenytoin was the second most commonly used ASM during hospitalization. Lacosamide replaced phenytoin as the second most commonly used ASM at discharge and during outpatient follow‐up.

### Outpatient follow‐up

For the patients started on ASMs during hospitalization, the first poststroke clinic visit was an average 49.7 (±31.7) days after the start of cEEG and 38.5 (±31.2) days after hospital discharge. By the time of the first poststroke clinic visit, eight patients (1.6% of the study cohort) had clinical seizures, all belonging to the group started on and continued on ASMs at discharge (2.8% of the ASM group). Seven of them had suffered an ASySs or had EAs on cEEG during admission. Five patients underwent an outpatient EEG, and 13 (6.9% of patients discharged on ASMs) saw an epileptologist during the follow‐up, including three patients with clinical seizures after hospital discharge. Additionally, 13 patients were weaned off the ASMs at the first poststroke clinic visit.

### Predictors of long‐term ASM continuation

To examine the factors predicting ASM use beyond the first poststroke clinic visit, only patients who were started on ASMs during hospitalization (*n* = 294) were used in these analyses. These patients were divided into two groups: those who were continued on ASMs beyond the first poststroke clinic visit (long‐term ASM continuation; *n* = 157) and those who were no longer on ASMs after the clinic visit (short‐term ASM use; *n* = 138). The descriptive statistics and the univariate logistic regression model comparing the two patient groups based on their ASM usage (short‐term vs. long‐term continuation) are presented in Table 2. Cortical involvement was related to greater odds of long‐term ASM continuation [OR 2.76 (95% CI 1.72–4.47)]. Additionally, patients experiencing SAH had lower odds of long‐term continuation compared to IS patients [OR 0.3 (95% CI 0.16–0.56)]. Electro‐clinical findings were a clear differentiating feature between the two groups. Among long‐term ASM continuation group, 56% had ASySs, compared to only 7% patients in short‐term ASM use group [OR 16.14 (95% CI 8.22–34.93)]. Epileptiform abnormalities were also more prevalent in long‐term ASM continuation group [OR 2.73 (95% CI 1.63–4.66)], as was a history of stroke [OR 3.74 (95% CI 1.65–9.63)]. Finally, admission to the NICU was associated with lower odds of long‐term continuation [OR 0.49 (95% CI 0.25–0.92)].

**Table 2:** Univariate analysis of demographic and clinical characteristics associated with long‐term ASM continuation.

	Long‐term ASM continuation (*n* = 156)	Short‐term ASM use (*n* = 138)	*p* values	Unadjusted odds ratio (95% CI)	*p* values
	*M* (SD)	*M* (SD)			
Age (years)	62.0 (14.2)	59.5 (13.7)	0.134	1.01 (1, 1.03)	0.134
NIHSS at admission	8.9 (8.3)	9.2 (8.5)	0.793	1 (0.96, 1.03)	0.792
Hospitalization duration (days)	13.1 (9.7)	14.4 (9.3)	0.256	0.99 (0.96, 1.01)	0.256
Time from discharge until follow‐up (days)	34.0 (27.7)	37.9 (31.7)	0.170	0.99 (0.99, 1)	0.172
Female	85 (54.5)	74 (53.6)	0.975	1.04 (0.65, 1.64)	0.882
Stroke type			<0.001		
Ischemic stroke	51 (32.7)	32 (23.2)		*Ref*	
ICH	75 (48.1)	44 (31.9)		1.07 (0.6, 1.9)	0.82
SAH	30 (19.2)	62 (44.9)		0.3 (0.16, 0.56)	<0.01
Cortex involved	108 (69.2)	62 (44.9)	<0.001	2.76 (1.72, 4.47)	<0.01
Initial mental status: Stupor/Coma	35 (22.4)	34 (24.6)	0.759	0.88 (0.52, 1.52)	0.657
Discharge destination: Home	58 (37.2)	46 (33.3)	0.571	1.18 (0.73, 1.92)	0.491
Patient admitted in NICU	123 (78.8)	122 (88.4)	0.042	0.49 (0.25, 0.92)	0.03
Discharging team			<0.001		
Neurology	77 (49.7)	41 (29.7)		*Ref*	
Med/Surg	16 (10.3)	6 (4.3)		1.42 (0.54, 4.21)	0.497
Neurosurgery	62 (40.0)	91 (65.9)		0.36 (0.22, 0.59)	<0.01
Seizures (convulsive or electrographic)^1^	87 (55.8)	10 (7.2)	<0.001	16.14 (8.22, 34.93)	<0.01
Epileptiform abnormalities on cEEG	64 (41.0)	28 (20.3)	<0.001	2.73 (1.63, 4.66)	<0.01
History of stroke prior to admission	26 (16.7)	7 (5.1)	0.003	3.74 (1.65, 9.63)	<0.01
Type of provider at follow‐up visit			0.022		
Neurologist	76 (48.7)	47 (34.1)		*Ref*	
APP	18 (11.5)	27 (19.6)		0.41 (0.2, 0.82)	0.013
Neurosurgeon	62 (39.7)	64 (46.4)		0.6 (0.36, 0.99)	0.046

Data presented as mean (standard deviation) or *N* (%).

^1^ Eight patients had seizures prior to the start of cEEG as well.

All the predictors associated with the outcome at least at the *p* < 0.05 level were added to the multivariable logistic regression model, with two exceptions. History of stroke was omitted due to sparsity in the nonoccurrence of the outcome variable (5%), though it was a significant univariate predictor. The type of discharge team was also omitted due to multicollinearity concerns with the follow‐up team variable. Based on the multivariable model (Table 3), the independent predictors of increased likelihood of long‐term ASM continuation were the presence of EAs on cEEG [OR 2.26 (95% CI 1.14–4.58)] and occurrence of an ASySs (convulsive or electrographic) [OR 20.31(95% CI 9.45–48.43)]. Admission to the NICU lowered the odds of long‐term ASM continuation [OR 0.37 (95% CI 0.15–0.92)]. A post hoc analysis showed that the 245 patients admitted to the NICU (accounting for 83% of patients started on ASMs) had a longer hospitalization duration [mean 14.7 days (±9.6)] than those not admitted to the NICU [8.6 days (±7.5)] (*p* < 0.001). However, there was no difference in time from hospital discharge to poststroke clinic visit for NICU and non‐NICU patients [37.1 (±30.3) vs. 29.5 (±25.9), respectively; (*p* = 0.102)]. Due to the above difference, a second multivariable model with an interaction term between NICU admission and hospitalization duration was created. However, the interaction was nonsignificant. To further explore NICU admission's effect on long‐term ASM usage, we also examined the relationship between admission to the NICU and ASM status at hospital discharge. Fewer NICU patients were discharged on ASMs (151/245; 62%) than non‐NICU patients (38/49; 78%) (*p* = 0.034).

**Table 3:** Multivariable logistic regression model for ASM continuance after clinic visit.

Variables	Adjusted odds ratio (95% CI)	*p* values
Age	1.01 (0.99, 1.04)	0.26
Stroke type		
Ischemic stroke	*Ref*	
ICH	1.79 (0.84, 3.89)	0.137
SAH	0.4 (0.16, 1.01)	0.053
Cortex involved	1.31 (0.67, 2.56)	0.433
Initial mental status		
Awake/Lethargy	*Ref*	
Stupor/Coma	0.77 (0.35, 1.67)	0.511
Patient admitted in NICU	0.37 (0.15, 0.9)	0.03
Follow‐up provider		
Neurologist	*Ref*	
APP	0.73 (0.28, 1.88)	0.519
Neurosurgeon	1.69 (0.82, 3.55)	0.16
Time from discharge until follow‐up (in days)	0.99 (0.98, 1)	0.249
Epileptiform abnormalities on cEEG	2.26 (1.14, 4.58)	0.021
Seizures (convulsive or electrographic)	20.31 (9.45, 48.43)	<0.01

The following variables were retained for multivariable modeling: age, stroke type, admission duration, cortical involvement, initial mental status, NICU admission, provider at follow‐up, time from stroke to follow‐up, clinical or electrographic seizure, and epileptiform abnormalities on cEEG. History of stroke was omitted due to sparsity in the nonoccurrence of the outcome variable (5%), though it was a significant univariate predictor. Discharge team was also omitted, due to multicollinearity concerns with the follow‐up team variable.

## Discussion

Almost one third (30.8%) of the 507 stroke patients with ASySs concerns in our single‐center, retrospective cohort study were continued on ASMs after the first poststroke clinic visit. They constitute more than half (53%) of the patients started on ASMs acutely. Factors like the stroke type, stroke severity, and cortical involvement, which are well‐known predictors of ASySs and epilepsy development after strokes,^1^, ^2^, ^3^, ^30^, ^31^ are not the primary drivers of long‐term ASM continuation. Hemorrhagic strokes may still influence ASM initiation as noted in Table 1. However, in the era of cEEG, electro‐clinical features like ASySs, convulsive or electrographic, and acute epileptiform abnormalities on EEG are the only independent predictors increasing the odds of long‐term ASM continuation.

The odds of patients with ASySs continuing ASMs for the long‐term was 20 times compared to those without ASySs. This decision was made, on average, more than 6 weeks after the initial concern for ASySs (49.7 ± 31.7 days from the start of cEEG). We find that in the absence of a clear definition of the “acute” period of ASM use after poststroke ASySs concerns, four of the five patients (82.5%) discharged on them continue their long‐term use beyond the poststroke clinic visit. Interestingly, almost half (*n* = 69; 44.2%) of the patients who continued on ASMs for the long‐term never had a convulsive or electrographic ASySs (Fig. 3). It is partly explained by the presence of EAs in the acute period. The odds of long‐term ASM continuation in patients with EAs on cEEG were more than twice that of patients without EAs. There is a clear association of EAs on cEEG with ASyS risk^14^ and, around 30%–35% of patients with EAs like LPDs also develop epilepsy.^32^, ^33^, ^34^ However, a quarter of long‐term ASM continuation patients (26.3%) did not have ASySs or EAs on cEEG after stroke. The reasons for ASM continuation in these patients are unclear and perhaps represent individual‐level decision‐making based on variables unaccounted for in our study.

The ASM discontinuation was significantly more likely to occur prior to, rather than after, hospital discharge (35.7% vs. 11.2%, respectively, of patients started on ASMs). NICU admission was a significant, independent predictor decreasing the long‐term ASM continuation odds by almost one third. It is despite a significantly higher percentage of NICU patients getting started on ASMs than non‐NICU patients. In contrast, NICU patients are significantly less likely to be discharged from hospital on ASMs than non‐NICU patients (62% vs. 78%, respectively). Combined, these two findings suggest a lower threshold of NICU caregivers to start their patients on ASMs, who usually have a poor mental status, a well‐known ASyS risk,^15^, ^35^ followed by swifter discontinuation. NICU caregivers are the earliest adopter and the most frequent user of cEEG monitoring. Close to 90% of the 105 patients with ASMs discontinuation prior to discharge lacked ASySs or EAs on their cEEG, suggesting cEEG’s influence on ASM management decisions. NICU caregiver’s familiarity and a deeper understanding of cEEG findings may aid their brisk ASM initiation and discontinuation decisions. None of the patients whose ASM was discontinued prior to hospital discharge had seizures in the follow‐up to the poststroke clinic. The follow‐up period in our study is too short to draw any conclusion about the safety of ASM discontinuation before hospital discharge. Prior evidence suggests that only 5%–7% of patients with acute brain injury lacking ASySs and EAs on cEEG develop epilepsy.^34^, ^36^

Lack of deprescribing and overtreatment remains a recalcitrant issue in epilepsy care.^37^, ^38^ ASySs account for 40% of all afebrile seizures with a lifetime risk of 3.6%,^39^, ^40^ and their diagnosis is more frequent in the cEEG era. They are often recurrent, with an estimated 20% risk of 30‐day mortality.^41^, ^42^ Therefore, ASM use during hospitalization to prevent ASySs recurrence is a prevalent practice.^8^ Almost all ASySs patients in our study were discharged on ASMs [97% (96/99); Fig. 3], consistent with the literature.^17^ By far, Levetiracetam was the most prescribed ASM in our patient cohort (Fig. 2), both acutely and as outpatient, consistent with the prevalent trend.^7^ Levetiracetam exhibits anti‐epileptogenesis potential in animal models.^43^ There is also some weak evidence suggesting it may prevent post‐traumatic epilepsy (PTE) after a high prophylactic dose.^44^ Although there is no definitive evidence of ASMs altering epileptogenesis in humans, the above findings inspire experts to argue that we may be missing an opportunity for clinically discovering the anti‐epileptogenesis potential of existing ASMs therapies.^45^ In this context, the continuation of ASM therapy in patients with ASySs or EAs beyond the acute period could be argued as a reasonable approach.

However, this leads to the vexing question of optimal duration of ASM therapy in these patients. In the absence of high‐quality evidence, expert recommendations range from 1 month to 3–12 months,^16^ and some recommend several years.^10^ cEEG influences ASM use beyond the acute period in current clinical practice. A recent study of SAH patients undergoing cEEG found that the ones with EAs, including seizures, are significantly more likely to continue ASMs beyond the standard prophylactic period.^46^ In prior smaller studies, we found 40%–80% of patients with ASySs and EAs on cEEG continuing ASMs for as long as 12–20 months.^32^, ^34^, ^36^ Although we did not analyze chronic ASM use, given the exponential growth in cEEG use^47^ and our finding that ASySs and EAs on cEEG significantly increase the odds of long‐term ASM continuation, a thorough investigation of current patterns of ASM use in this patient population is clearly warranted. If a patient is discharged on ASMs, our results suggest that the poststroke care providers may be reluctant to discontinue ASM at the clinic visit. The level of experience and expertise with ASM management may differ among various neurological care providers. Post‐acute symptomatic seizure (PASS) clinics served by epileptologists with a major clinical interest and practice focus in cEEG monitoring could bridge this gap in clinical care and scientific knowledge.^48^

We provide data on posthospital discharge follow‐up in a large cohort of stroke patients with ASySs concerns during hospitalization. The analysis of cEEG influence on the long‐term ASM continuation, along with their acute use, attempts to fill a large knowledge gap that remains unaddressed despite close to two decades of conspicuous use of this diagnostic modality. Our findings shed light on predictors of the long‐term ASM continuation in stroke patients with ASySs concerns. As a single‐center study, our findings may reflect some influence of institutional practices, including cEEG monitoring. The use of cEEG for acute stroke patients is a clinical decision and dependent on several factors, which may differ among individual providers, teams, and institutions. While combining stroke type does not find them a predictor of long‐term ASM continuation, future studies should investigate specific stroke types as each type may have unique predictors of ASM use. While more than 85% of eligible patients were seen in the poststroke clinic (Fig. 1), we lack data on a small number of patients who did not follow‐up with our health system. Despite a large and well‐characterized cohort, we could not evaluate the differential impact of convulsive versus electrographic seizures and stroke history on long‐term ASM continuation due to a small number of patients under these categories in the short‐term ASM use group.

Although our retrospective observational study does not lend itself to ASM management recommendations in stroke patients with ASySs concerns, it provides new information about the extent of ASM use in these patients. We find that ASMs are more often discontinued prior to hospital discharge, especially in patients admitted to the NICU, than at the poststroke clinic visit. Close to a third of these patients continue on ASMs for the long term. Stroke‐related factors do not influence this practice based on multivariable regression analysis. Instead, ASySs and EAs on cEEG significantly increase the odds of long‐term ASM continuation. However, almost a quarter of patients undergoing long‐term ASM continuation lack ASySs or EAs after stroke and underscores the need for thorough, prospective studies. With acute brain injuries like stroke accounting for a major proportion of morbidity, mortality, and health‐care burden, a multicenter collaborative approach is required to develop evidence‐based guidelines of ASM use in these patients.

## Conflict of Interest

Vineet Punia, Ryan Honomichl, Pradeep Chandan, Lisa Ellison, Nicolas Thompson, Adithya Sivaraju, Irene Katzan, Pravin George, Chris Newey, and Stephen Hantus report no relevant conflict of interest.

## Supporting information

**Table S1**. Clinical characteristics of patients started on ASM with no seizures or epileptiform abnormalities (EAs). ^1^NIHSS available for 94 patients (61 in ASM discontinued before discharge and 33 in ASM continued on discharge).
