# Supplementary material for: Long‐term continuation of anti‐seizure medications after acute stroke
Source: Ann Clin Transl Neurol. 2021 Aug 6;8(9):1857–66. doi: 10.1002/acn3.51440 (PMC8419404; doi:10.1002/acn3.51440)
Supplement: Supplementary file 1 — Table S1. Clinical characteristics of patients started on ASM with no seizures or epileptiform abnormalities (EAs). 1NIHSS available for 94 patients (61 in ASM discontinued before discharge and 33 in ASM continued on discharge). [file ACN3-8-1857-s001.docx]

Supplemental Table 1: Clinical characteristics of patients started on ASM with no seizures or epileptiform abnormalities (EA)

|  | Patients started on ASM with no seizures/EAs  (n = 146) | ASM discontinued before discharge (n = 91) | ASM continued at discharge  (n = 55) | ***p* values** |
| --- | --- | --- | --- | --- |
| Age (years) | 58.7 (13.6) | 58.3 (14.1) | 59.4 (13.0) | 0.65 |
| NIHSS at admission^1^ | 7.5 (7.2) | 8.9 (8.3) | 5.0 (5.8) | 0.02 |
| Hospitalization duration (days) | 13.5 (9.0) | 15.0 (9.8) | 11.1 (6.9) | 0.01 |
| Female | 76 (52.1) | 46 (50.5) | 30 (54.5) | 0.73 |
| Stroke Type |  |  |  | <0.001 |
| Ischemic stroke | 33 (22.6) | 20 (22.0) | 13 (23.6) |  |
| ICH | 59 (40.4) | 26 (28.6) | 33 (60.0) |  |
| SAH | 55 (37.7) | 45 (49.5) | 10 (18.2) |  |
| Cortex involved | 71 (48.6) | 38 (41.8) | 33 (60.0) | 0.04 |
| NSGY procedure | 75 (51.4) | 46 (50.5) | 29 (52.7) | 0.86 |
| Hemorrhagic conversion | 4 (2.7) | 3(3.3) | 1 (1.8) | 1 |
| Initial Mental Status: Stupor/Coma | 26 (17.8) | 21 (23.1) | 5 (9.1) | 0.008 |
| Patient Admitted in NICU | 124 (84.9) | 80 (87.9) | 44 (80.0) | 0.23 |
| Discharging Team |  |  |  | 0.61 |
| Neurology | 44 (30.1) | 30 (33.0) | 14 (25.5) |  |
| Med/Surg | 9 (6.2) | 5 (5.5) | 4 (7.2) |  |
| Neurosurgery | 93 (63.7) | 56 (61.5) | 37 (67.2) |  |

^1^NIHSS available for 94 patients (61 in ASM discontinued before discharge and 33 in ASM continued on discharge).
